# Properties of Concrete with Tire Derived Aggregate Partially Replacing Coarse Aggregates

**DOI:** 10.1155/2015/863706

**Published:** 2015-05-25

**Authors:** Gideon Siringi, Ali Abolmaali, Pranesh B. Aswath

**Affiliations:** ^1^Materials Science and Engineering Department, University of Texas at Arlington, Arlington, TX 76019, USA; ^2^Lhoist North America, 3700 Hulen Street, Fort Worth, TX 76107, USA; ^3^Civil and Environmental Engineering Department, University of Texas at Arlington, Arlington, TX 76019, USA

## Abstract

Tire derived aggregate (TDA) has been proposed as a possible lightweight replacement for mineral aggregate in concrete. The role played by the amount of TDA replacing coarse aggregate as well as different treatment and additives in concrete on its properties is examined. Conventional concrete (without TDA) and concrete containing TDA are compared by examining their compressive strength based on ASTM C39, workability based on ASTM C143, splitting tensile strength based on ASTM C496, modulus of rupture (flexural strength) based on ASTM C78, and bond stress based on ASTM C234. Results indicate that while replacement of coarse aggregates with TDA results in reduction in strength, it may be mitigated with addition of silica fume to obtain the desired strength. The greatest benefit of using TDA is in the development of a higher ductile product while utilizing recycled TDA.

## 1. Introduction

It is estimated that, in the USA, each person discards one car tire per year. With a population of over 300 million people, it indicates that every year there are a total of 300 million tires that need to be disposed [1–3]. In recent years, some innovative ways of using these tires have been developed. Some of these include tire derived fuel (TDF) for cement kilns and boilers [1] and tire derived aggregates (TDA) used as raw materials for civil engineering projects [3]. However not all tires are consumed in these beneficial ways and the scrap tires that remain are disposed in various legal and illegal means (disposal of tires in an unpermitted area). Whole tires are difficult to landfill because they tend to float back to the surface with time. Stockpiles of scrap tires result in public health, environmental, and aesthetic problems in addition to being fire hazards [2].

It is with this environmental concern that the US government through the Environmental Protection Agency (EPA) encourages more studies on methods of recycling tires [2]. One beneficial use of tires that has been proposed is tire derived aggregate (TDA) as a replacement of mineral aggregates in concrete [4, 5]. However, none of the studies have elucidated in any detail the beneficial aspects of TDA and the mechanism by which the properties of TDA reinforced concrete differ from traditional concrete. In this study we hope to detail the properties of concrete where some of the coarse aggregate (rock) is replaced with TDA.

It is hoped that TDA can be a lightweight substitute for mineral aggregates as its density is less than half of that of mineral aggregate. Mineral aggregates have a unity density ranging from 100 to 130 lb/ft^3^ (1600–2080 kg/m^3^) while TDA's unit density ranges from 40 to 45 lb/ft^3^ (640–720 kg/m^3^) [6].

## 2. Experimental Procedure

**Figure 1:**
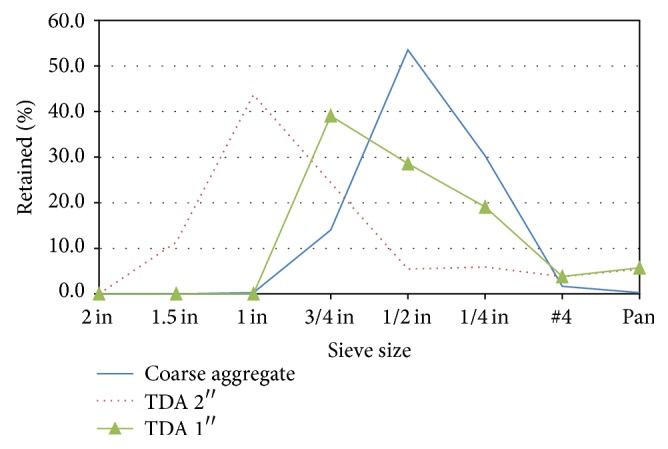
Coarse aggregate and TDA particle distribution comparison.

The major raw materials used in this experiment were coarse aggregates with maximum size of 1.5 in (38.1 mm) and fine aggregates with maximum size of 0.187 in (4.75 mm) both meeting ASTM C33 requirements. Two sizes of tire derived aggregate (TDA) were used, one with a maximum size of 1 in (25.4 mm) and the other with a maximum size of 2 in (50.8 mm). Both sizes of TDA came from the same batch and only sieving was done to differentiate the two sizes. The designation of 2^″^ size and 1^″^ size only refers to the maximum size of TDA particle but in total, the TDA would contain all sizes of particles below that size as shown in the particle size distribution obtained through sieve analysis in Figure 1.

Other raw materials were tap water from local municipality and commercially available Type III Portland cement with a fineness of 98% passing a 325 mesh (45 *μ*m sieve) and a Blaine of 540 m^2^/kg. The silica fume used in this study was compacted silica fume which is pozzolanic material composed of highly refined silicon dioxide in noncrystalline form. Commercially available epoxy, PC Products, PC-Concrete 600 mL Concrete Bonding Agent, was used.

Concrete proportioning was done following the Absolute Volume Method as described by Portland Cement Association [7]. The 28-day compressive strength of over 4500 psi (31 MPa) was targeted while the Portland cement content was based upon water/cement (w/c) ratio of between 0.55 and 0.60. The actual batch compositions in terms of weight are shown in Table 1. The batches were prepared, mixed, and cured following ASTM C192 [8]. At the completion of mixing, the concrete was deposited in a wheel barrow and slump test was carried out following ASTM C143 [9].

The 6 in × 12 in (150 mm × 300 mm) cylinders and 20 in × 6 in × 6 in (510 mm × 150 mm × 150 mm) beams were cast. The cylinders were used to test for compressive strength following ASTM C39 [10] and splitting tensile test following ASTM C496 [11]. The 20 in × 6 in × 6 in (510 mm × 150 mm × 150 mm) concrete beams were used to test for flexural strength following ASTM C78 [12].

**Figure 2:**
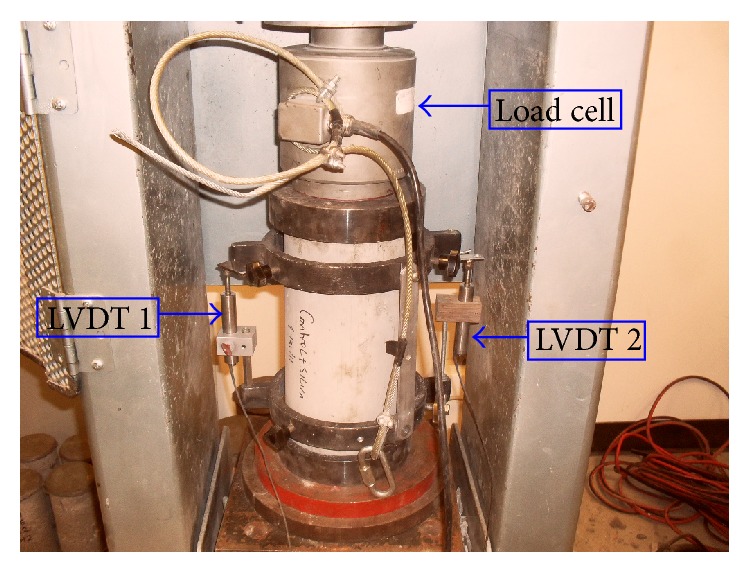
Compressive test (ASTM C39) setup.

From each batch several cylinders and beams were cast. The molded cylinders and beams were cured at 80°F (26°C) and relative humidity of about 100%. One set of three cylinders or beams was tested after 7 days and another set of three after 28 days for every batch. ASTM C39 test method was followed for compression tests where the applied load was measured using a load cell and displacement was measured using two linear variable differential transformers (LVDTs) all of which were connected to a computer system. The computer system included a Vishay Scanner, Model 5100B, and a laptop computer with Strainsmart5000 software. The two LVDTs were attached on a tailored cylinder which was screwed to the body of the concrete cylinder to measure displacement of the concrete directly as shown in Figure 2. The LVDTs used were Omega's LD621-5 with a Range of 0 to 10 mm (0 to 0.4′′). The data collected was load (lb) and displacement in inches from each LVDT. In all the calculations, the average displacement from the two LVDTs was used.

The splitting tensile test followed the ASTM C496 and flexural strength followed ASTM C78, the applied load was measured using a load cell, and displacement of the testing machine head was measured using Novotechnik position transducers (TR 100) with a range of 0–100 mm.

Pull-out tests were also performed based on ASTM 234. The purpose was to determine the bond strength between concrete and deformed steel reinforcing bars due to the adhesion of the paste to the steel, the friction between the steel and the concrete, and the bearing of the concrete against the lugs of the deformed steel bars. The direct pull-out is used to test the bond strength of reinforcing rods in concrete. ASTM 234 recommends using the direct pull-out test for determining the bond strength developed between the concrete and reinforcing steel. The moulds used in this experiment were the 6 in × 12 in (150 mm × 300 mm) cylinders and #4 steel bars.

The direct pull-out test method consisted of a #4 steel bar embedded through a cylindrical concrete specimen. The specimens had the steel bar embedded at the depth of  4 in (101.6 mm) of the full length of the cylindrical specimen (12 inches (300 mm)). The concrete was constrained and the steel rod was pulled from one end of the specimen. The bond strength of the concrete is determined from the force applied to the rebars divided by the interfacial contact area of the rebar bonded region.

One of the objectives of the study was to establish an optimum amount of TDA that can be used to replace coarse aggregates without significantly compromising the strength of the concrete. The starting point was to replace 100% of coarse aggregate and then the amount of TDA was reduced until an optimum amount was obtained. To this end a control batch (batch with no TDA) was first prepared. The mix composition is shown in Table 1. The compressive strength of the control batch was used as a standard from which the strength of concrete where TDA replaced some or all of the coarse aggregates (TDA batch) was compared to. All process factors in TDA batches are held constant except for the replacement of coarse aggregates (rock) with an equal volume of TDA.

Once the compressive strength of the control batch and the TDA batch was determined, if there was a big drop in strength, the amount of TDA was dropped and the experiment repeated again. The different amounts of coarse aggregates replaced with an equal amount of TDA (by volume) were 100%, 17%, 10%, and 7.5%. Once the optimum amount of TDA was obtained, all the other tests (ASTM C78, ASTM C496, ASTM C143, and ASTM 234) were done on the batch with the optimum amount of TDA.

The optimum amount of TDA was determined to be between 7.5 and 10%. At this percentage, several other options were explored to improve the strength further in the TDA batches. One of them was to reduce the size of TDA from a maximum of 2^″^ (50.8 mm) to 1^″^ (25.4 mm). The TDA particle distribution is shown in Figure 1 in comparison with coarse aggregates. The main consideration of size of TDA was to use a TDA size close to the size of the mineral aggregates to be replaced and also consider the cost of the aggregates: the finer the TDA is, the more expensive it becomes.

**Figure 3:**
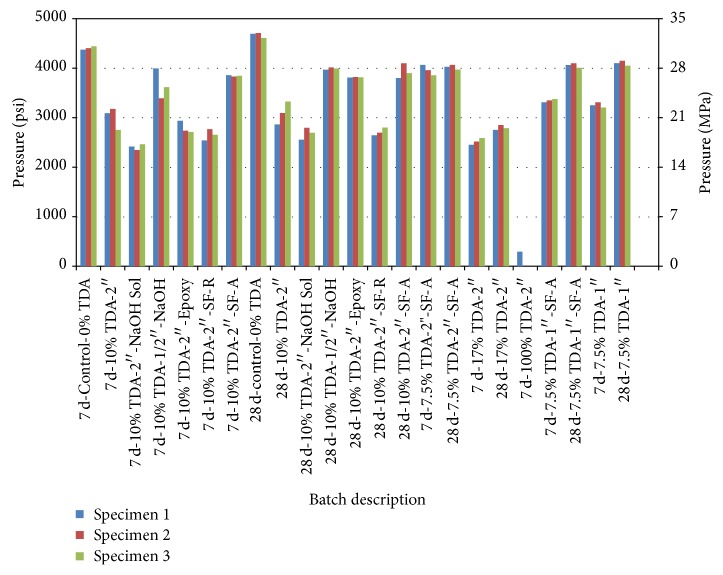
Summary of compressive test results with loading up to concrete failure at 7 days and 28 days. The batch designation is as shown in Table 2.

Other options explored to improve strength of the concrete with TDA were treatment of TDA particles with sodium hydroxide (NaOH) solution, epoxy, and incorporation of silica fume into the concrete batch. Earlier studies had suggested that treatment with NaOH enhanced bonding with concrete [4] and hence was one of the methods attempted. The second method involved using a 2-part epoxy with the hope that this would improve bonding between TDA and concrete and the last method attempted was the incorporation of silica fume which has been shown to improve the strength of concrete [13]. Silica fume was used in two ways, first it replaced 20% cement and later an amount equal to 20% of cement was added to the concrete without replacing any cement. All compressive strength results are shown in Figure 3.

[Table tab2]

NaOH was used in two ways. At first, a third of the total water required was set aside and used to prepare a 1-Molar NaOH solution where the TDA was immersed in the solution for 30 minutes prior to introduction of both the solution and the TDA to the mixer. When this method did not yield good results as shown in Figure 3, a second method was attempted. Here, 1-Molar NaOH solution was prepared and TDA was immersed in the solution for 24 hours after which the TDA was introduced to the mixer while it was still wet but the NaOH solution was discarded. The rest of the procedure remained the same as described earlier.

When the two-part epoxy was used, TDA was placed in a container and the two parts of epoxy were added to it, mixed, and then introduced to the mixer immediately well before the epoxy started to set. The rest of the procedure was then followed as described in the earlier section.

## 3. Results and Discussion

### 3.1. Workability

Workability is defined in terms of the amount of mechanical work or energy required to produce full compaction of the concrete without segregation [14]. Workability also refers to concrete consistency, flowability, mobility, pumpability, compactibility, finishability, and harshness. Workability of a freshly mixed concrete was evaluated through slump measurement as outlined in ASTM C143 [9]. The slump test is considered to be a measure of the shear resistance of concrete to flowing under its own weight.  Table 3 shows representative slump measurements for the different batches that were prepared.

Incorporation of TDA into concrete results in an increase of the slump by an average of 1 inch at the same water/cement ratio when compared to the control. Higher slump implies better workability when shaping fresh concrete into desired shapes during construction. Aiello and Leuzzi [15] made the same observations of improved workability when they investigated the properties of various concrete mixtures at fresh and hardened state obtained by a partial substitution of coarse and fine aggregate with different volume percentages of waste tires rubber particles, having the same dimensions of the replaced aggregate. The size range of the rubber particles they used was between 10 mm and 25 mm. Topçu and Bilir [16] made similar observation of improved workability with introduction of rubber into concrete. However, Toutanji [17] recorded slump measurements showing that workability decreased with introduction of rubber.

It was found out that one should consider silica fume as a cementitious material in calculating amount of water required when silica fume is incorporated in concrete if the same workability is to be achieved in the absence of either a water reducer or superplasticizers. This conclusion is from the observed reduction in slump with the introduction of silica fume (Table 3).

At low doses of 3 percent or less, silica fume serves to liquefy the concrete by fitting in between the cement grains due to their small size that they displace water, which becomes free to help with the flowability of the concrete. In effect, it becomes its own water reducer. But when you add more and more silica fume, up to the neighborhood of 5 percent of cementitious material, the surface area of the silica fume begins to outweigh its water displacement function, surface forces begin to have a strong effect, and water reducer, superplasticizer, or both must be added to overcome the need for more water [18].

### 3.2. Compressive Strength

Strength is defined as a measure of the stress required to fracture a material. Figure 3 is a summary of compressive strength of various batches with different amounts of TDA in comparison with regular concrete (control) both at 7 days and 28 days. When all the coarse aggregate is replaced with TDA (100% TDA-2^″^), the TDA concrete developed only 8% of the strength of that of the control concrete at 7 days. This was a very drastic drop and it was concluded that only very little amount of TDA can be used as a substitute for coarse aggregates. These results are consistent with Siddique and Naik [19] who had also reported approximately a 85% reduction in compressive strength when coarse aggregate is fully replaced by coarse crumb rubber chips.

Replacing 17% of coarse aggregates (17% TDA-2^″^) with an equal volume of TDA, all other factors remaining constant saw a 45% drop in strength at 7 days and 40% drop at 28 days in comparison with the control concrete. This drop was still considered large and therefore unacceptable. It was then decided to drop the quantity of TDA further in order to improve on the strength. These results confirm those of Huang et al. [20] who recorded a 45% compressive strength reduction when 15% of coarse aggregate were replaced by rubber chips.

When 10% of coarse aggregate (10% TDA-2^″^) was replaced with an equal volume of TDA, there was a slight mitigation of the properties, with a drop of 28.6% and 33.8% at 7 days and 28 days, respectively, when compared to the control batch (Control-0% TDA). This drop in compression strength is still high but it was deemed that it would be impractical to further reduce the amount of TDA. At this point ways of improving concrete strength were sought.

Also a summary of compressive strength is shown in Figure 3 when NaOH solution, epoxy, and silica fume were incorporated into concrete containing TDA. NaOH treatment of rubber before introduction of TDA and NaOH solution (10% TDA-2^″^-NaOH Sol) into the concrete did not improve the bonding between the concrete constituents and the rubber chips and in fact the overall compressive strength declined when compared with the samples with an equal amount of TDA but with no NaOH treatment as seen from Figure 3.

This is the opposite of results obtained by Pelisser et al. [21] and Segre et al. [4] whose results indicated that NaOH increases rubber particle's surface hydrophilicity hence improving bonding between the rubber and concrete. However, when the TDA was treated with NaOH solution before being added to the mixer, the solution discarded (7.5% TDA-1^″^-NaOH) saw the strength of the concrete being equal to that with equal amount of TDA but no treatment with NaOH (7.5% TDA-1^″^).

Possible negative effect of NaOH could have been increased solubility of gypsum in the cement leading to flash set in the concrete. NaOH addition may also result in undesirable morphology and nonuniformity of hydration products in the pastes, thus reducing cement strength. Addition of NaOH generally decreases ettringite formation [22]. Ettringite is a hydration product formed through the reaction of tricalcium aluminate (C_3_A) and gypsum in the presence of water. Formation of ettringite slows down the hydration of C_3_A by creating a diffusion barrier around C_3_A therefore allowing for more time for tricalcium silicate (C_3_S) to hydrate. C_3_S is the cement compound that is responsible for strength development.

After 7 days of curing the 2-part epoxy did not result in improvement of strength but had a significant impact after 28 days. The 28-day strength was 18% less compared to the control batch. This showed that the epoxy improved the TDA concrete by about 23% when compared to TDA concrete without epoxy at 28 days. Since no improvement was noted at 7 days (early strength) when using epoxy, this method was considered unattractive.

Replacing part of cement with silica fume did not have any positive effect on strength as seen from Figure 3 (10% TDA-2^″^-SF-R) but addition of silica fume (7.5% TDA-2^″^-SF-A) on top of the cement into had a positive effect on concrete strength. At 10% coarse aggregate replacement with TDA and addition of silica fume equal to 20% of cement, the drop in strength was 12.4% at 7 days and 16.5% at 28 days compared to the control batch. Pelisser et al. [21] observed that compressive strength was reduced by 14% at 28 days, in comparison to the conventional concrete when 10% sand aggregate was replaced by recycled tire rubber. Both conventional rubber and rubber modified with alkaline activation were used with silica fume addition to improve mechanical properties. Zheng et al. [23] found a 22.3% decrease in strength with 15% rubber replacements by the volume of the coarse aggregate at 28 days. However, these results are a big improvement from the results recorded by Schimizze et al. [24]. Schimizze et al. record a strength loss of about 50% by the addition of 5% rubber by weight.

The overall compressive strength of about 3900 psi (≈27 MPa) was also deemed acceptable and falls within the range of structural concretes. Mindess et al. [14] define structural concretes as one with compressive strength between 17–63 MPa (2465−9135 psi). The results of improved strength when using silica fume agree with Güneyisi et al. [25] who showed that the addition of silica fume into the matrix improved the mechanical properties of the rubberized concretes and diminished the rate of strength loss.

Silica fume (SF) functions in a concrete as a highly efficient pozzolan, that is, it reacts chemically with the calcium hydroxide produced by the hydration of the Portland cement to form calcium silicate hydrates (C-S-H) which bind the concrete together. Silica fume is highly reactive due to the high proportion of noncrystalline SiO_2_ and the large surface area [13].

Silica fume can be used in concrete in two ways: as an addition (generally 8–15% by mass of cement), to enhance properties of the fresh and/or hardened concrete or as a partial cement replacement (5–10% by mass of cement) to maintain the 28-day compressive strength at lower cement content (with associated environmental benefits) while reducing the heat of hydration and improving durability [13]. From the results in Figure 3 the former would be preferred when using TDA.

The functions of silica fume in Portland cement concrete are twofold, both physical and chemical in nature. Physically, there are three major attributes for silica fume. Because the silica fume particles are much smaller than the cement particles—with a surface area in the neighborhood of 20,000 m^2^/kg—they can “pack” between the cement particles and provide a finer pore structure. This property is particularly important because it is likely that TDA could be increasing the void content due to poor bonding to the concrete resulting in the low strength in concretes with TDA. The final strength of the concrete is in a large part a function of the amount of compaction; a small increase in void content (or decrease in relative density) will lead to a large decrease in strength. In the early stages of hydration, silica fume can help accelerate the hydration process, because its tiny particles provide nucleation sites for hydration.

In the nucleation process, a silica fume particle provides a site on which material in solution can “nucleate” or “center,” which helps the material precipitate sooner than it might otherwise do. And once it precipitates, the concentration of that material in solution is reduced, which tends to get more material into solution from elsewhere, speeding the process. Silica fume can dramatically reduce bleeding as it introduces a lot of surface area into the mix, which in turn helps hold the water in place.

Chemically, if time and moisture are allowed to do their job, silica fume has a very strong pozzolanic reaction, so that when the cement grains hydrate and generate calcium hydroxide, the silica fume will react with that and create more calcium silicate hydrate. In this instance, more space is filled up within the concrete, which gives much more strength, and improves resistance to intrusion from a number of factors. These benefits include radically reduced permeability to water and reduced diffusivity to chloride ions.

A further reduction of quantity of TDA to 7.5% replacement had only a marginal effect even with incorporation of silica fume into the batch as seen from the results in Figure 3. The results elucidate that the batch with TDA developed 87.6% of the control batch strength at 28 days. At 7 days, the batch with TDA showed 8.5% reduction in compressive strength compared with the control. This was only a 3.4% improvement from the batch where 10% coarse aggregate was replaced with TDA.

From these results it was concluded that small amounts of TDA with a maximum size of 2 inches in the range of 7.5–10% can be used to replace coarse aggregates in concrete whose compressive strength is about 4000 psi (28 MPA). To achieve this compressive strength, strength enhancing materials like silica fume would need to be used. This recommendation is about half of what Khatib and Bayomy [26] recommend. Khatib and Bayomy recommend that rubber contents should not exceed 20% of the total aggregate volume.

However, if the size of TDA is reduced, the results in Figure 3 show that one can achieve a compressive strength of up to 4000 psi without using strength enhancing materials like silica fume. The choice of TDA size would then depend on cost considerations, that is, the cost of further reduction of TDA size versus the cost of the strength enhancing materials.

### 3.3. Concrete Ductility

Apart from the positive environmental effect of using TDA as a lightweight replacement for mineral aggregates, it was hoped that TDA would improve some other properties of concrete like ductility. Rubber which is the source of TDA under same stress conditions would deform much more than mineral aggregates since it has a lower elastic modulus but the material would deform at almost constant volume as Poisson's ratio for TDA is approximately 0.48 [27]. Another significant difference between TDA and mineral aggregates is that individual particles of TDA are more deformable and tend to bend more easily than sand and gravel particles.

At high stress level, the strain no longer remains proportional to the applied stress, and it becomes permanent; that is, it would not be reversed if the specimen is unloaded. This strain is called plastic or inelastic strain. Typically under compression, concrete appears to show inelastic strain at fracture of the order of 2 × 10^−3^ [28]. Ductility is defined as the ability of a material to deform easily upon the application of a load or as the ability of a material to withstand plastic deformation without rupture. Ductility may also be thought of in terms of bendability and crushability. Ductile materials show large deformation before fracture. The lack of ductility is often termed brittleness.  Table 4 compares ductility of different types of concrete in terms of inelastic strains.

**Figure 4:**
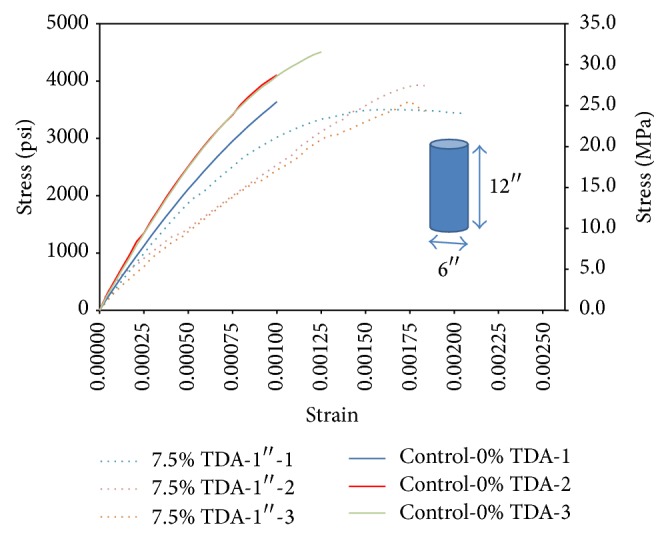
Stress versus strain comparison between control concrete and TDA concrete at 28 days.

Two concretes (7.5% TDA-1^″^ and Control-0% TDA) are compared in Figure 4 using stress-strain curves. In Figure 4 the batch with no TDA is labeled Control-0% TDA 1/2/3 and the batch in which TDA replaced 7.5% of coarse aggregates is labeled 7.5% TDA-1^″^ 1/2/3-No silica. On an average, the deformation the concrete with TDA can sustain before failure as shown in Figure 4 and Table 4 is higher than those without TDA even though they fail at slightly lower strength. Zheng et al. [23], Toutanji [17], Aiello and Leuzzi [15], Khaloo et al. [29], and Eldin and Senouci [30] made a similar observation. The concrete with TDA exhibited improved postcracking behavior, showing a good energy absorption and ductility and the concrete with TDA did not demonstrate the typical brittle failure, but rather a ductile, plastic failure mode.

**Figure 5:**
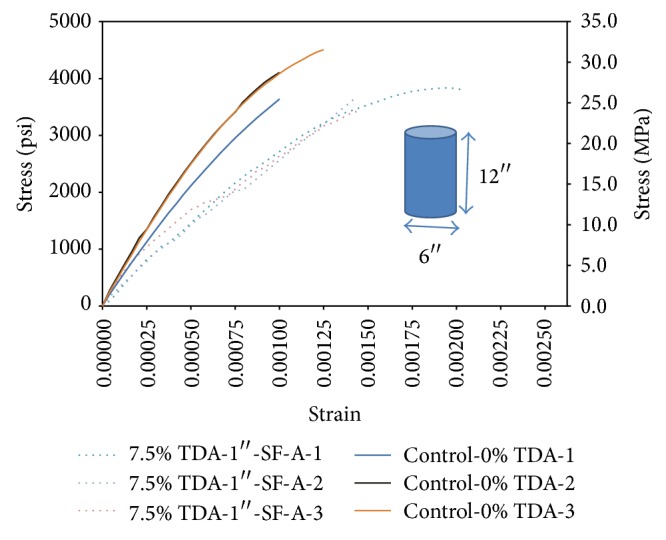
Stress versus strain comparison between control concrete and TDA concrete with silica fume at 28 days.

Figure 5 has the same stress-strain comparison between the control batch (Control-0% TDA) and TDA batch but this time with addition of silica fume (7.5% TDA-1^″^-SF-A). Silica fume is noted to have improved the consistency of TDA concrete and its strength but appears to have had a negative effect on the amount of strain the concrete can sustain before failure when comparing 7.5% TDA-1^″^-SF-A and 7.5% TDA-1^″^ total strain in Table 4.

Ductility can also be quantified in terms of percent elongation or reduction in length in a tensile or compressive test, respectively. The percent elongation/reduction provides additional information on the deformational characteristics of the material and is an indicator of ductility. The average total displacement (reduction in original length) for the Control-0% TDA, 7.5% TDA-1^″^, and 7.5% TDA-1^″^-SF-A was 0.0136 in (0.3461 mm), 0.0228 in (0.58 mm), and 0.0198 in (0.5038 mm), respectively. Since the original cylinder length was 12 in (300 mm), the percent elongation for the three types of concrete is 0.11%, 0.19%, and 0.17% for Control-0% TDA, 7.5% TDA-1^″^, and 7.5% TDA-1^″^-SF-A, respectively.

### 3.4. Concrete Toughness

The energy required to break the material, the product of force and distance, is represented by the area under the curve of the stress-strain plot. The term modulus of toughness is a measure of this energy.

Calculation of area under the curves in Figures 4 and 5 is summarized in Table 5. TDA is shown to improve concrete toughness but the effect is diminished if silica fume is used. Huang et al. [20] and Toutanji [17] also found that rubberized concrete had very high toughness when they replaced coarse aggregate with rubber chips.

### 3.5. Modulus of Elasticity

The modulus of elasticity is defined as the ratio between the stress and the reversible strain. It is a measure of stiffness of a component. The elastic modulus of concrete in compression varies from 14 × 10^3^ to 40 × 10^3^ MPa (2 × 10^6^ to 6 × 10^6^ psi) [28]. The significance of the elastic limit in structural design lies in the fact that it represents the maximum allowable stress before the material undergoes permanent deformation.

However, due to concrete nonlinearity, three methods are used to compute the modulus giving rise to three types of moduli. These are the tangent modulus given by the slope of a line drawn tangent to the *σ*-*ε* curve at any point on the curve, secant modulus given by the slope of a line drawn from the origin to a point on the curve corresponding to a 40% stress at failure load, and chord modulus given by the slope of a line drawn between two points on the *σ*-*ε* curve. The chord modulus was used in the calculations by shifting the base from the origin to correct the slight concavity observed at the beginning of the *σ*-*ε* curve up to a point about 40% of the stress at failure.

Table 5 summarizes the computed results for elastic modulus. From Table 5 it is found that using of TDA in concrete would lower the elastic modulus of concrete by about 20%. Güneyisi et al. [25] also in their study indicated that there is a large reduction in the strength and modulus values with the increase in rubber content in concrete. A steep slope of the stress-strain curve, thus a high modulus of elasticity, means that a greater force is required to stretch bonds and hence higher binding energy. A lower modulus of elasticity in concrete containing TDA could then signify low binding energy (weak bonds) between TDA particles and the rest of the concrete components. It could also mean a higher porosity in concrete with TDA.

However if a stress of 3000 psi (20.68 MPa) is applied to each material, the concrete without TDA deforms elastically to a maximum of 0.001^″^/in while the concrete with TDA would deform elastically to a minimum of 0.00125 in/in as shown in Figures 4 and 5. Therefore concrete with TDA would deform elastically 20% more compared with concrete without TDA.

### 3.6. Behavior of Concrete under Uniaxial Compression

Generally, the stress-strain curve shows a linear, elastic behavior up to about 30% of the ultimate strength, *f*_*c*_′, because under the short-term loading the microcracks in the interfacial transition zone remain undisturbed. For stresses above this point, the curve shows a gradual increase in curvature up to about 0.75*f*_*c*_′ to 0.9*f*_*c*_′ then it bends sharply (almost becoming flat at the top) and finally descends until the specimen is fractured [28].

**Figure 6:**
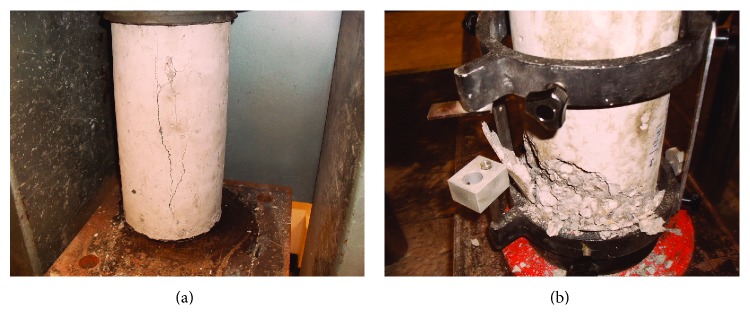
Control-0% TDA concrete failure patterns. The fracture line was generally parallel to the loading direction and failure was catastrophic.

**Figure 7:**
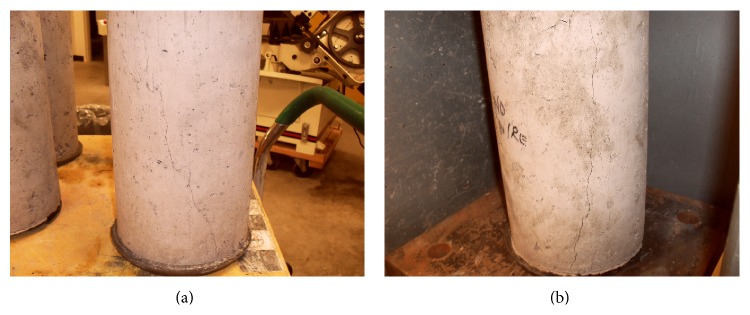
7.5% TDA-1^″^ concrete failure patterns. The fracture line was generally at an angle to the loading direction.

Concrete contains void spaces of various sizes and shapes in the matrix and microcracks at the interfacial zone therefore failure modes vary with the type stress. In uniaxial compression, as stress increases, cracks are initiated within the matrix; their number and size increase progressively. Eventually cracks in the matrix and the interfacial transition zone (shear-bond cracks) eventually join up, and generally a failure surface develops at about 20° to 30° from the direction of the load.  Figure 6 shows failure modes for control concrete (Control-0% TDA). Generally the fracture line is straight and runs through the specimen. However, as seen from Figure 7, TDA concrete (7.5% TDA-1^″^) fracture line can be up to 45° from the direction of the load, not straight, and does not run through the specimen. This may explain why the TDA concrete does not have a brittle failure like the control concrete in Figure 6. Khaloo et al. [29] also demonstrated a significant decrease in the brittle behavior of concrete with increasing rubber content and unlike plain concrete; the failure state in rubberized concrete occurred gently and uniformly and did not cause any separation in the specimen.

The difference between the two concretes was the amount of course aggregates. One had part of coarse aggregates replaced by TDA. The difference in behavior of the two concretes is thought to have been caused by changes in the interfacial transition zone characteristics due to the different size, shape, and surface texture of the aggregate particles therefore affecting the concrete strength and failure modes. It is also believed that due to the smooth surface of TDA particles, a weak physical bond between TDA particle and the hydrated cement particle is formed which is responsible for the lower strength of TDA containing concrete.

Two characteristics of aggregates have an important influence on proportioning concrete mixtures. These are grading (particle size distribution) and nature of the particle (shape, porosity, and surface texture). The aggregates are predominantly responsible for the unit weight, elastic modulus, and dimensional stability of the concrete. These properties of concrete depend to a large extent on the bulk density and strength of the aggregate, which in turn are determined by physical rather than chemical characteristics of the aggregate. Grading is important for attaining an economical mixture because it affects the amount of concrete that can be made with a given amount of cement and water.

### 3.7. Splitting Tensile Strength of Cylindrical Concrete

Table 5 summarizes the results for splitting tensile strength. From the results it seen that the average splitting strength for the control batch was 466 psi (3.2 MPa) while the batch with 7.5% of coarse aggregates replaced with an equal volume of TDA was 525 psi (3.6 MPa). This represents 12.7% improvement in splitting tensile strength at 28 days.

Another important consideration in the testing of concrete mixes is the percent a particular strength parameter compares to the 28-day design compressive strength, *f*_*c*_′. The standard by which all concrete strengths are compared is that of *f*_*c*_′ for the identical mix, cured under the identical conditions, and at the same age. For the two batches, the control batch developed an average of 10.4% of *f*_*c*_′ while the batch with 7.5% of coarse aggregates replaced with an equal volume of TDA developed 13.1% of *f*_*c*_′. *f*_*c*_′ was taken to be 4500 psi (≈31 MPa) for control concrete and 4000 psi for TDA concrete.

At 7 days (early strength), the average splitting strength for the control batch was also 466 psi (3.2 MPa) while the batch with 7.5% of coarse aggregates replaced with an equal volume of TDA was 431 psi (3.0 MPa). This represented 7.6% drop in splitting tensile strength for the batch with TDA compared with the control batch at 7 days. Generally, splitting tensile strength is used in the design of structural lightweight concrete members to evaluate the shear resistance provided by concrete and to determine the development length of reinforcement. From the results at 28 days, it was concluded that, in this respect, the TDA concrete would perform satisfactorily or superior to the control concrete in the long time.

**Figure 8:**
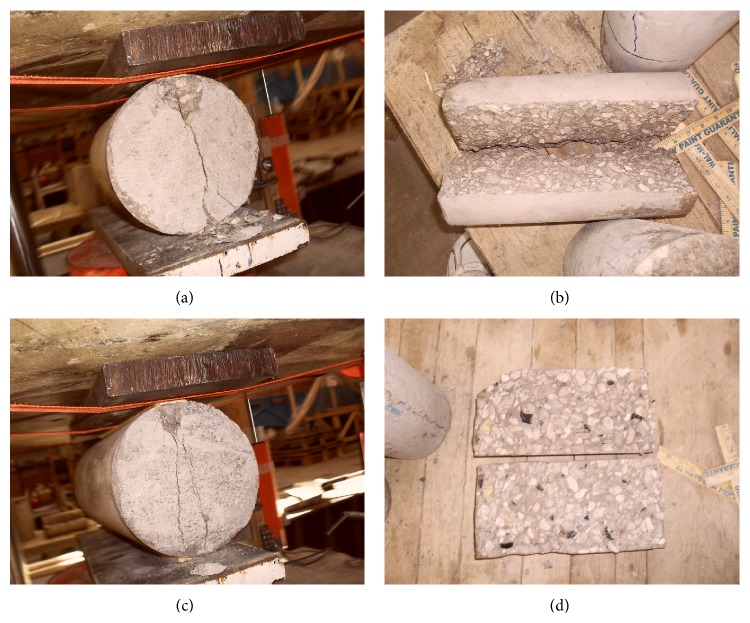
((a) and (b)) Failure pattern for Control-0% TDA. Single fracture line cutting through the specimen is noted. (c) 7.5% TDA-1^″^ during splitting tensile test showing multiple fracture lines. (d) Distribution of TDA in the 7.5% TDA-1^″^ concrete.

As seen from Figure 8, the control concrete (Control-0% TDA) developed a single fracture line which ran through the specimen while the TDA concrete (7.5% TDA-1^″^) developed multiple fracture lines which were not joined as loading was increased probably due to the presence to TDA particles between the fracture lines. This may explain the superior performance noted for TDA concrete in terms of ultimate splitting tensile test.

### 3.8. Flexural Strength (Modulus of Rupture) of a Concrete Beam

Table 5 summarizes the results for the flexural strength of a concrete beam with loading at the third point at 7 and 28 days after casting. The tests at 28 days of flexural strength of a concrete beam with loading at the third points show the control (Control-0% TDA) having an average modulus of rupture of 570 psi (3.93 MPa). The batch in which 7.5% of coarse aggregate was replaced by TDA with silica fume addition (7.5% TDA-1^″^-SF-A) had a modulus of rupture of 480 psi (3.31 MPa) while the batch which did not include silica fume (7.5% TDA-1^″^) developed a modulus of rupture of 535 psi (3.69 MPa). This represented a drop of 15.8% and 6.1% for TDA concrete with and without silica fume, respectively. At 7 days, this drop was 22.9% and 8.6% drop from the control batch, respectively.

**Figure 9:**
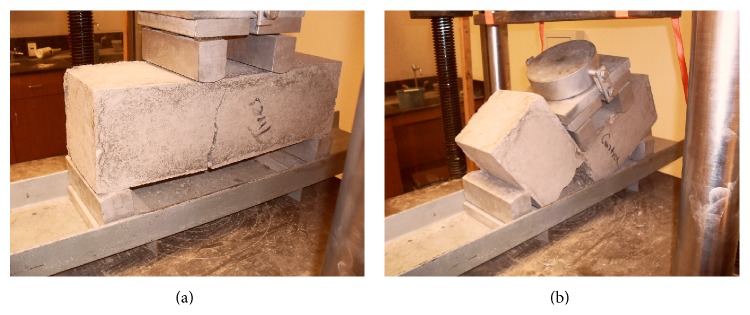
(a) Failure pattern for 7.5% TDA-1^″^ concrete beam. The beam did not fail into 2 halves and fracture line was not parallel to loading direction. (b) Failure pattern for Control-0% TDA concrete beam. The beam failed into 2 halves and fracture line was parallel to loading direction.

Figure 9 shows the fracture lines for TDA concrete and control concrete during flexure test, respectively. The fracture line for control concrete was found to be straight (follow the loading direction) and the specimen failed completely into two halves. The TDA concrete fracture line did not follow the loading direction and the specimen did not fail completely into two halves. This was found to be due to the cracks not being able to cut through the TDA particles. This could be an advantage in structures in avoiding catastrophic failures.

**Figure 10:**
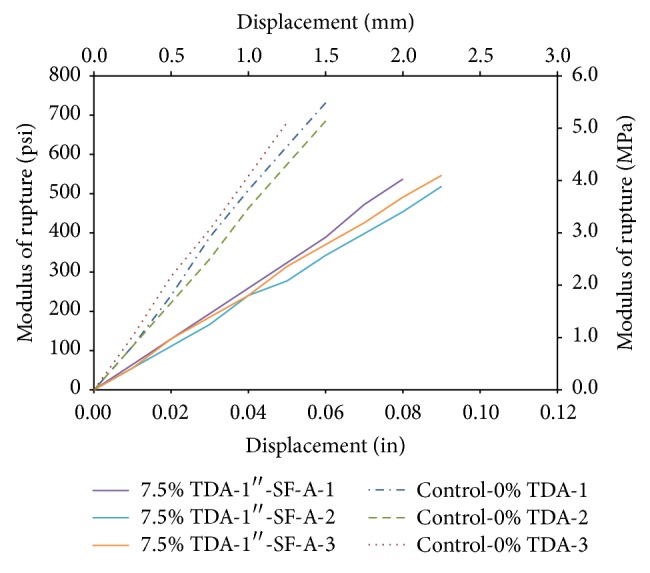
Comparison of modulus of rupture between the control concrete (Control-0% TDA) and TDA concrete with silica fume (7.5% TDA-1^″^-SF-A).

A plot of modulus of rupture (psi/MPa) against displacement (in/mm) is shown in Figure 10 comparing the control concrete and TDA concrete. The displacement for TDA concrete is average 50% higher than the control concrete even though the modulus of rupture is low. This is a good indication of improved concrete ductility since a more ductile material would undergo higher displacement (deformation) before failure.

### 3.9. Pull-Out Test

**Figure 11:**
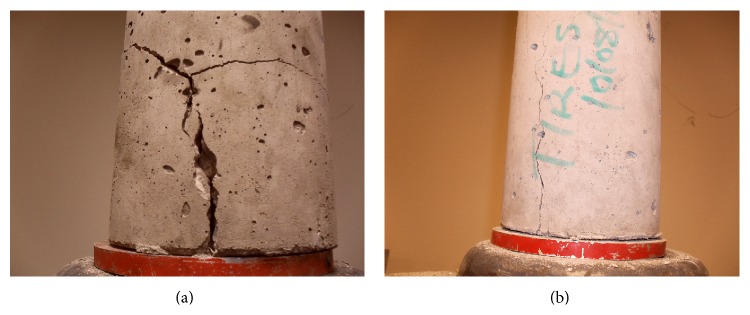
(a) Failure pattern for Control-0% TDA concrete, (b) 7.5% TDA-1^″^ concrete failure pattern during pull-out test. Wide and multiple cracks are noted in the Control-0% TDA concrete while a single crack forms in 7.5% TDA-1^″^ concrete.

The calculated peak bond stress results from the pull-out test are as shown in Table 5. Overall there was a difference of 2% between the averages of the control specimen and the specimen with TDA. This difference is small and can be considered to be within experimental error. This implies that using TDA in concrete would not affect negatively the bond strength of reinforcing rods in concrete. Observation of failure patterns in Figure 11 for control concrete and TDA concrete shows that TDA would prevent widening of cracks and hence prohibit catastrophic failure.

**Figure 12:**
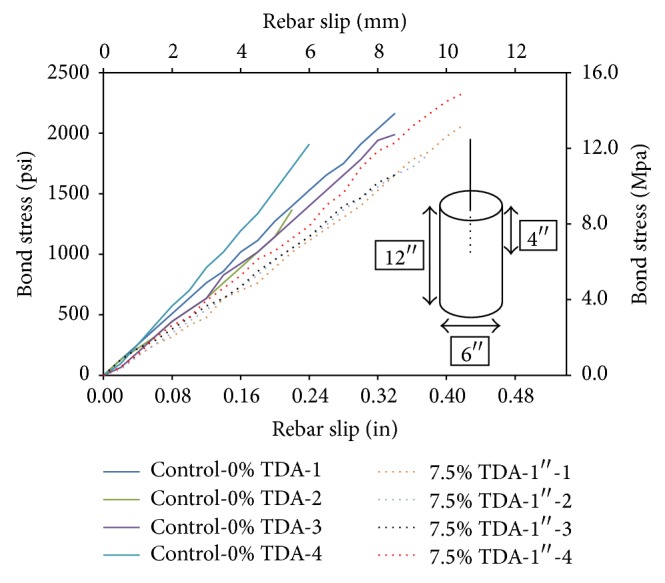
Comparison of bond stress against rebar slip for Control-0% TDA and 7.5% TDA-1^″^ at 28 days.

A plot of the bond stress against rebar slip is shown in the Figure 12 for the two types of concrete. Average rebar slip for the four control concrete specimen is 0.29 in (7.4 mm) while that of TDA is 0.39 in (9.9 mm) representing an increase of 37%. Since the calculated bond strength represents the adhesion of the paste to the steel, the friction between the steel and the concrete, and the bearing of the concrete against the lugs of the deformed steel bars it therefore means that TDA would lower these properties and hence the increased rebar slip observed.

## 4. Conclusion

Small amounts of waste tires (TDA) in the range of 7.5% to 10% can be used in concrete with a target compressive strength of up to 4000 psi (≈28 MPa) but strength enhancing materials like silica fume need to be used. As TDA increase, the compressive strength drops. At 7.5% of TDA replacing coarse aggregates, this drop is found to be approximately 10% compared to the control concrete if silica fume is added into the mixture. However, this amount of strength can also be achieved without using strength enhancing materials (silica fume) if the top TDA size is lowered from 2 in to 1 in.

Using TDA to substitute for mineral aggregates lowers the modulus of elasticity of concrete by about 20% but increases the concrete toughness and ductility. However silica fume, as much as it increases compressive strength and consistency, has a negative effect to ductility. Workability of fresh concrete with TDA is slightly better as it has a slump of averagely 1 in higher compared to the control concrete. However if a stress of 3000 psi is applied to each material, the concrete without TDA deforms elastically to a maximum of 0.001 in/in while the concrete with TDA would deform elastically to a minimum of 0.00125 in/in. Therefore concrete with TDA would deform elastically 20% more compared with concrete without TDA.

TDA lowers the modulus of rupture of concrete but it increases displacement up to 50% (improved concrete deformation) during loading. The splitting tensile strength improves by 12.7% with introduction of TDA into concrete. The bond strength of the TDA concrete is not significantly different from that of the control concrete but TDA improves postcracking behavior of the concrete as noted from the pull-out tests.

## Figures and Tables

**Table 1:** Mix proportions for various concrete batches prepared for testing compressive strength to determine amount of TDA to be used.

Component, lb (kg)	Batch number											
	1	2	3	4	5	6	7	8	9	10	11	12
Cement	92.5 (42)	92.5 (42)	92.5 (42)	92.5 (42)	92.5 (42)	92.5 (42)	92.5 (42)	92.5 (42)	92.5 (42)	92.5 (42)	92.5 (42)	92.5 (42)

Silica fume								18.5 (8.4)	18.5 (8.4)	18.5 (8.4)	18.5 (8.4)	

Rock (coarse aggregates)	393.6 (179)		326.7 (148)	354.3 (161)	354.3 (161)	354.3 (161)	354.3 (161)	354.3 (161)	354.3 (161)	364.1 (165)	364.1 (165)	364.1 (165)

Sand (fine aggregates)	259.2 (118)	259.2 (118)	259.2 (118)	259.2 (118)	259.2 (118)	259.2 (118)	259.2 (118)	259.2 (118)	259.2 (118)	259.2 (118)	259.2 (118)	259.2 (118)

Water	51.2 (23)	51.2 (23)	51.2 (23)	51.2 (23)	51.2 (23)	51.2 (23)	51.2 (23)	51.2 (23)	51.2 (23)	51.2 (23)	51.2 (23)	51.2 (23)

TDA		118.8 (54)	24.3 (11)	14.3 (6.5)	14.3 (6.5)	14.3 (6.5)	14.3 (6.5)	14.3 (6.5)	14.3 (6.5)	10.3 (4.8)	10.3 (4.8)	10.3 (4.8)

2-part epoxy							✓					

NaOH					✓	✓						

Total	796 (361)	522 (237)	754 (342)	771 (350)	771 (350)	771 (350)	771 (350)	771 (350)	790 (358)	796 (361)	796 (361)	777 (352)

**Table 2 tab2:** Batch number, designation, and description.

Batch number	Designation	Description
1	Control-0% TDA	Control mix design, no TDA used

2	100% TDA-2′′	100% replacement of coarse aggregate with TDA of size 2′′ (50 mm)

3	17% TDA-2′′	17% replacement of coarse aggregate with TDA of size 2′′ (50 mm)

4	10% TDA-2′′	10% replacement of coarse aggregate with TDA of size 2′′ (50 mm)

5	10% TDA-2′′-NaOH Sol	10% replacement of coarse aggregate with TDA of size 2′′ (50 mm). TDA dissolved in NaOH solution and both TDA and NaOH solution included in batch

6	7.5% TDA-1′′-NaOH	10% replacement of coarse aggregate with TDA of size 2′′ (50 mm). TDA dissolved in NaOH solution for 24 hours but solution not included in the batch

7	10% TDA-2′′-Epoxy	10% replacement of coarse aggregate with TDA of size 2′′ (50 mm). TDA treated with 2-part epoxy before mixing

8	10% TDA-2′′-SF-R	10% of coarse aggregate replaced with an equal volume of TDA size 2′′ (50 mm) and 20% of cement replaced with silica fume

9	10% TDA-2′′-SF-A	10% of coarse aggregate replaced with an equal volume of TDA size 2′′ (50 mm) and silica fume equal to 20% of cement added to the mix

10	7.5% TDA-2′′-SF-A	7.5% of coarse aggregate replaced with an equal volume of TDA size 2′′ (50 mm) and silica fume equal to 20% of cement added to the mix

11	7.5% TDA-1′′-SF-A	7.5% of coarse aggregate replaced with an equal volume of TDA size 1′′ (25 mm) and silica fume equal to 20% of cement added to the mix

12	7.5% TDA-1′′	7.5% of coarse aggregate replaced with an equal volume of TDA size 1′′ (25 mm)

**Table 3:** Slump measurements.

	Water/ cement ratio	Slump (in)	Slump (mm)
Control-0% TDA	0.55	0.5	12.7
7.5% TDA-2′′-SF-A	0.55	0.25	6.4
7.5% TDA-1′′-NaOH	0.55	1.5	38.1
Control-0% TDA	0.60	2.25	57.2
7.5% TDA-1′′	0.60	3.5	88.9
7.5% TDA-1′′-SF-A	0.60	2	50.8

**Table 4:** Total strain deformation comparison for the different types of concrete.

	Control-0% TDA	7.5% TDA-1′′	7.5% TDA-1′′-SF-A
Total strain (*ε*)			
Specimen 1	0.00100	0.00204	0.00204
Specimen 2	0.00125	0.00183	0.00142
Specimen 3	0.00100	0.00183	0.00146
Average total strain (*ε*)	0.00108	0.00190	0.00164
Average elongation, in (mm)	0.0136 (0.3461)	0.0228 (0.5800)	0.0198 (0.5038)
% elongation	0.11%	0.19%	0.17%

**Table 5:** Calculated elastic modulus, splitting tensile strength, flexural strength, peak bond stress, and calculated area under stress-strain plots for the control and TDA concrete with and without silica fume.

Component	Control-0% TDA	7.5% TDA -1′′	7.5% TDA -1′′-SF-A
	Elastic modulus, psi (MPa)		
At 28 days	3.35*E* + 06 (2.31*E* + 04)	2.72*E* + 06 (1.88*E* + 04)	2.69*E* + 06 (1.85*E* + 04)
At 7 days	2.69*E* + 06 (1.85*E* + 04)	2.37*E* + 06 (1.63 + 04)	2.13*E* + 06 (1.85*E* + 04)

	Splitting tensile strength, psi (MPa)		
At 28 days	466 (3.2) ± 7.9%	525 (3.6) ± 7.2%	
At 7 days	466 (3.2) ± 17%	431 (2.97) ± 6%	

	Flexural strength (modulus of rupture), psi (MPa)		
At 28 days	570 (3.93) ± 10.7%	535 (3.69) ± 2.7%	480 (3.31) ± 6.9%
At 7 days	602 (4.2) ± 10%	550 (3.8) ± 7%	464 (3.2) ± 12%

	Pull-out test, peak bond stress, psi (MPa)		
At 28 days	1929 (13.3) ± 12.3%	1890 (13.0) ± 14.3%	

	Calculated area under stress-strain plots representing concrete toughness		
At 28 days	75.7 ± 31%	215 ± 29%	154 ± 15%
